# The reliability and validity of the Japanese version of the Levels of Emotional Awareness Scale (LEAS-J)

**DOI:** 10.1186/1751-0759-5-2

**Published:** 2011-01-31

**Authors:** Tetsuya Igarashi, Gen Komaki, Richard D Lane, Yoshiya Moriguchi, Hiroki Nishimura, Hiromi Arakawa, Motoharu Gondo, Yuri Terasawa, Corbet V Sullivan, Motonari Maeda

**Affiliations:** 1 Department of Psychosomatic Research, National Institute of Mental Health, National Center of Neurology and Psychiatry, 4-1-1 Ogawa-higashi Kodaira-City, Tokyo, 187-8553, Japan; 2 Department of Psychiatry, University of Arizona, 1501 N. Campbell Ave., Tucson, AZ 85724-5002, USA; 3 Centre for Advanced Research on Logic and Sensibility, Keio University, 2-15-45 Mita, Minato-ku, Tokyo 108-8345, Japan; 4 Language Education and Research Center, Kyushu Sangyo University, 2-3-1 Matsukadai, Higashi Ku, Fukuoka 813-8503, Japan; 5 College of Art and Design, Joshibi University of Art and Design, 1900 Asamizodai, Sagamihara-City, Kanagawa 228-8538, Japan

## Abstract

**Background:**

The Levels of Emotional Awareness Scale (LEAS) was developed to assess five levels of emotional awareness: bodily sensations, action tendencies, single emotions, blends of emotion, and combinations of blends. It is a paper and pencil performance questionnaire that presents 20 emotion-evoking scenes. We developed a Japanese version of the LEAS (LEAS-J), and its reliability and validity were examined.

**Methods:**

The LEAS-J level was independently assessed by two researchers who scored each response according to the LEAS scoring manual. High inter-rater reliability and internal consistency were obtained for the LEAS-J. Measures were socioeconomic status, LEAS-J, Toronto Alexithymia Scale-20 (TAS-20), Interpersonal Reactivity Index (IRI), and NEO Five-Factor Inventory (NEO-FFI). TAS-20, IRI and NEO-FFI were the measures used to explore the construct validity of LEAS-J, as it was predicted that higher scores on the LEAS-J would be related to fewer alexithymic features, greater empathetic ability, and a greater sense of cooperation with others. Questionnaires were completed by 344 university students.

**Results:**

The criterion-referenced validity was determined: a significant negative relationship was found with the externally-oriented thinking scores of TAS-20, and positive relationships were found with fantasy, perspective taking, and empathic concern on IRI and with extraversion, openness to experience, and agreeableness on NEO-FFI.

**Conclusions:**

Consistent with our expectations, the findings provide evidence that the LEAS-J has good reliability and validity. In addition, women had significantly higher scores than men on LEAS-J, showing that the gender difference identified in the original LEAS was cross-culturally consistent.

## Background

The conscious awareness of one's own emotions is a prerequisite for emotional intelligence, including the conscious regulation of one's own emotional states and expressive behaviors [1,2]. Higher levels of emotional awareness are also associated with greater physical and mental health [3].

Thus, a reliable and valid method is needed for assessment of awareness of emotions, particularly one that is applicable in a variety of cultural contexts. Lane & Schwartz [4] proposed a theoretical construct that divides emotional awareness into 'levels' based on the cognitive- developmental theory of Piaget [5]. Subsequently Lane et al. [6] developed the Levels of Emotional Awareness Scale (LEAS), for which good reliability and validity have been reported. The levels are as follows: (Level 0) Non emotion; (Level 1) Awareness of physiological cues; (Level 2) Awareness of action tendencies; (Level 3) Conveying a single specific emotion; (Level 4) Conveying two or more differentiated emotions; and (Level 5) Conveying two or more differentiated emotions for two or more persons. The LEAS consists of 20 scenarios that elicit feelings such as sadness, anger, fear, and happiness and blends of these feelings. The characters in each scenario are oneself (Self) and another person (Other). The participants are requested to explain in short essay style their expectation of how the Self and Other would feel in reaction to the scenario.

A number of methods, such as self-report questionnaires and structured interviews, have been developed for assessing difficulty in identifying and describing specific emotional states [7-9]. Self-reporting has serious limitations, however, when testing individuals with a limited and undifferentiated capacity for describing their emotional experience, namely, alexithymic subjects. It is questionable whether, when completing the items, these patients are sufficiently aware of their own difficulties to accurately rate themselves on their difficulty in emotional awareness and expression of their own feelings. In contrast to such self-reported measurements, examinees do not have to assess their own abilities regarding emotional awareness when using the LEAS; their descriptions are rated by examiners who place them into the appropriate levels of emotional awareness. Thus, LEAS is a performance-based measure for evaluating such an emotional capacity that is scored by trained raters who judge the written responses according to a strictly defined scoring structure.

In applying the LEAS to other countries, such as Japan, with cultures that may be quite different than that of the United States, it is important to take into account that the scenarios, which describe situations focusing on interpersonal relationships, need to be modified to fit the appropriate cultural context. Further, because emotional processes are influenced by cultural differences in attention and perception, cultural regulation of emotions should be considered when examining the level of emotional awareness [10]. With this in mind, the Japanese version of the LEAS was developed for possible future research on cross-cultural aspects of levels of emotional awareness. Thus, the present study was conducted to assess the reliability and validity of our newly developed Japanese version of the LEAS, the LEAS-J.

## Methods

### Subjects

The LEAS-J was given to 380 Japanese students, 344 (90.5%) of whom completed the questionnaires. They ranged in age from 18-38 y.o. [mean age (SD) = 20.13 (1.64)], and were recruited from two coeducational and one female-only university, all located in urban, middle class areas [males; n = 121, 18-26 y.o., mean age (SD) = 19.98 (1.42), female; n = 223, 18-38 y.o., mean age (SD) = 20.23 (1.74)]. The greater ratio of females to males resulted, in part, from the fact that women were recruited at all three sites, whereas men were recruited from only two.

The aim of the study was explained in a classroom setting. The students were told that any student who registered to participate would be paid a small fee for completing the questionnaires at home and for sending them back by mail. Because it was too time-consuming in the classroom setting to complete the test battery that included the numerous short essays required by LEAS, the participants were requested to complete them at home. They were informed in writing that their privacy would be completely protected and that no participant would be identified when the results of the study were published. This study was approved by the local ethics committee of the National Center of Neurology and Psychiatry.

### Measures

#### Socioeconomic status

The participants reported the occupations of their parents, and the 123 reported occupations were divided into a hierarchy of high, middle, and low class, based on the occupational prestige scores for Japan proposed by Tsuduki [11]. For example, high for medical doctors, low for workers with short term or daily contracts, and middle for office workers, public employees, and part-time workers who did the same type of work. Middle class workers were in the majority. Of the participants, five reported that their parents were "jobless", and no-answer was given for 13 fathers and 186 mothers, most of whom were housewives.

#### Development of LEAS-J

The LEAS is a performance-based measure of the levels of emotional awareness [4,6] (Table 1). Some of the scenarios were deemed unsuitable to the Japanese cultural context and were changed to comparable scenarios with the help of our American co-author (C.S.), who has lived in Japan for over 20 years, then evaluated by the original author (R.D.L.), who accepted them as having the same level of emotional content and likely to elicit a comparable response. For example, scenario 1 is a situation intended to invoke anger: The original scenario is of someone asking a neighbor to repair a piece of furniture at home, which would be quite uncommon in Japan. It was changed to a situation in which someone asks bystander to charge his car battery at parking lot. Both situations should invoke the identical emotion, anger. The Japanese version of the LEAS, including the revised scenarios, was back-translated into English by a person proficient in both English and Japanese. Finally, working with the original author, we confirmed that there were no major differences in the content of the original and back-translated versions of LEAS, except for Scenario 18. Because we felt the original scenario would be inconceivable in Japan, it was completely rewritten, as shown in Table 2.

**Table 1:** Examples of answers to an LEAS-J scenario

Scene1:	A person asks you to charge the battery of his/her car because it went dead. As you work to connect the cord, your thumb is injured. How would you feel? How would the person who asked you to charge the battery feel?	
	
Answer examples	Self	Other

Level 0	Why did that person ask me to do this?	If I had not asked him to charge it, it might not have happened.
Level 1	This hurts...	I'd feel sick to my stomach.
Level 2	How unlucky I am to have been caught up in this situation. It's unfortunate.	I feel awkward.
Level 3	I feel slightly sad because I have failed again.	I am afraid that the person will stop working and will not help me charge it.
Level 4	I feel it is unlucky. It should run after properly being charged. I have mixed feelings of disappointment, resignation, and anger.	I feel sorry, guilty, and regretful to have made the request.

Note: A Level 5 score is assigned to a scenario when both Self and Other are scored at Level 4 and are not identical with regard to emotional content.

**Table 2:** Changes from LEAS in LEAS-J scenarios

Scenario	Degree of Change	Change
1	minor	Replacing a battery / thumb is injured rather than repairing furniture / hit thumb with hammer
3	minor	Husband (wife) gently rubs your shoulder rather than loved one gives you a back rub
7	minor	Spouse has lived far away for a long time rather than sweetheart gone for several weeks
9	minor	Post office rather than bank
10	minor	Fire engines around the corner near your home rather than fire trucks parked near your home
14	minor	Quite deteriorated cavities rather than several cavities
16	minor	salty foods / salty noodles rather than Fatty foods / pizza
18	major	LEAS: You sell a favorite possession of your own in order to buy an expensive gift for your spouse. When you give him/her the gift, he/she asks if you sold the possession to buy it.
		LEAS-J: One of your family members has been hospitalized and now is going to be discharged. The hospital has strict regulations. A nurse says that they can not accept anything as a token of gratitude because the hospital has strict regulations. But you strongly wish to give them a token of gratitude because you are so grateful for their care.

#### The 20-item Toronto Alexithymia Scale

The Japanese translation of the 20-item Toronto Alexithymia scale (TAS-20) developed by Komaki, et al. [12] was used. Alexithymia is a concept proposed by Sifneos [9] as characteristic of patients with psychosomatic disorders and is associated with impaired verbal and nonverbal recognition of emotion [7,8,13]. The TAS-20 is based on the assumption that alexithymia is a continuous variable in the general population. TAS-20 is a self-reported questionnaire that consists of 20 items, with three subscales that measure characteristics of alexithymia. The subscales are (a) difficulty in identifying feelings (DIF); (b) difficulty in describing feelings to others (DDF); and (c) externally-oriented thinking (EOT). The Japanese version of the TAS-20 has good reliability and validity [12,14].

#### The Interpersonal Reactivity Index

The Interpersonal Reactivity Index (IRI) is a self-report questionnaire that measures the empathetic ability of the participants [15,16]. Each scale measures a distinct component of empathy; 1) Fantasy (FS), emotional identification with characters in books, films etc.; 2) Perspective Taking (PT), cognitively taking the perspective of another; 3) Empathic Concern (EC), feeling emotional concern for others; and 4) Personal Distress(PD), negative feelings in response to the distress of others. Emotional awareness and empathy to others have been reported to be positively correlated [17]. The Japanese version of the IRI has good concurrent validity and reliability [18].

#### NEO-Five Factor Inventory

The NEO-Five Factor Inventory (NEO-FFI), an abridged version of the NEO Personality Inventory, is one of the standard measures of the big five factor model of personality structure [19]. The five major domains (factors) of personality measured by this 60-item scale are as follows: Neuroticism (N), Extraversion (E), Openness to Experience (O), Agreeableness (A), and Conscientiousness (C). The relation between alexithymia and this five-factor model of personality was reported in previous studies [14,20-22]. The Japanese version of the NEO-FFI has good reliability and validity [23]. The answer format is a 5-point Likert-type scale (0-4), ranging from ''Strongly disagree'' (0) to ''Strongly agree'' (4). Scores are summed totals and have a range of 0-48 for each of the five personality domains.

### LEAS-J Scoring Procedure

LEAS-J was scored by two authors (T.I., H.N.), working independently, who classified the emotion-related words attributed to Self and Other for each scenario. Before scoring the protocols, training was undertaken by both scorers who discussed the results of pilot testing until accurate scores were achieved, based on the LEAS scoring manual [24].

Classification was according to the LEAS scoring manual [24]. We used the original glossary of words, translated into Japanese, at each level as follows: (Level 0) non emotion (e.g., Level 0 included no response given to an item or the description of a thought or impression that reflected an act of cognition without any indication of the emotional reaction that followed from the cognitive act); (Level 1) awareness of physiological cues; (Level 2) awareness of action tendencies; (Level 3) conveying a single specific emotion; (Level 4) conveying two or more differentiated emotions. Each subject received separate scores (0-4) for their descriptions of the feelings of Self and Other. The Total score for each item was the highest of the two (Self and Other) scores, except in the case where both Self and Other received level 4 scores; level 5 is scored when an item is scored 4 for both Self and Other and if the reactions of the Self and Other are clearly different from each other [24]. The score range is 0-80 for Self and Other and 0-100 for Total.

### Emotion-related word count survey

To extract emotion-related words from the data of the LEAS-J, we used the KHCoder, a Japanese corpus analysis software [25] that highlights selected words and automatically calculates word counts.

### Software and statistical significance

SPSS ver.11.0 was used for all statistical analysis. Statistical significance was set at p < 0.05, two-tailed.

## Results

Intra-class correlation coefficients [ICC (2, 1)] were calculated to determine the inter-rater reliability of each item and subscale of the LEAS-J (Table 3). The results were .59 ~ .87 for Self, .36 ~ .79 for Other, and .32 ~ .82 for Total. Although they were low for scenario 1, the correlation coefficients for the summed scores were sufficiently high (Self = .88; Other = .87; Total = .90). Therefore, we did an individual analysis of the scores of each rater to assess inter-rater reliability for the scores of Self, Other, and Total. Descriptive statistics for LEAS, TAS-20, IRI, and NEO-FFI are shown in Table 4.

**Table 3:** Between rater intraclass correlation coefficients [ICC(2, 1)] for the LEAS-J subscales

	Self	Other	Total
scene 1	.63	.36	.32
scene 2	.77	.79	.75
scene 3	.65	.79	.65
scene 4	.80	.76	.70
scene 5	.76	.66	.67
scene 6	.66	.73	.67
scene 7	.80	.73	.74
scene 8	.76	.65	.72
scene 9	.82	.65	.59
scene 10	.87	.66	.81
scene 11	.69	.54	.50
scene 12	.59	.69	.65
scene 13	.81	.53	.64
scene 14	.85	.75	.82
scene 15	.73	.53	.64
scene 16	.67	.78	.67
scene 17	.74	.60	.59
scene 18	.59	.52	.48
scene 19	.68	.73	.61
scene 20	.80	.73	.65
sum	.88	.87	.90

**Table 4:** Descriptive statistics of the LEAS-J, TAS-20, IRI, and NEO-FFI

	range	*M*	(*SD*)
[LEAS-J]			
Self	9.50-71.50	40.79	(10.98)
Other	11.00-63.50	36.76	(9.53)
Total	17.00-74.00	50.05	(9.38)
[TAS-20]			
DIF	0.00-28.00	11.06	(6.08)
DDF	2.00-22.00	14.34	(3.96)
EOT	8.00-31.00	18.66	(4.02)
sum	10.00-72.00	44.06	(9.84)
[IRI]			
FS	3.00-28.00	18.07	(5.76)
PT	0.00-27.00	15.98	(4.19)
EC	3.00-28.00	17.51	(4.54)
PD	0.00-28.00	16.48	(5.22)
[NEO-FFI]			
N	8.00-52.00	33.34	(7.98)
E	9.00-49.00	29.83	(7.24)
O	10.00-43.00	28.96	(5.67)
A	6.00-44.00	26.10	(6.35)
C	9.00-52.00	30.36	(7.06)

### Reliability of LEAS-J

Cronbach's α coefficients were calculated for the Self, Other, and Total responses to the 20 scenarios as independently scored by two raters (T.I, H.N.). Table 5 shows the results of the reliability of LEAS-J. The Chronbach's α coefficients were .82 for Self, .77 for Other, and .83 for Total.

**Table 5:** The *α *coefficients of the LEAS-J subscales

	Self	Other	Total
α score	.82	.77	.83

### Differences in LEAS-J scores by sex and socioeconomic status

T-tests were used for comparisons between males and females. The LEAS-J scores of females were higher than those of males on all subscales (Table 6). Differences of TAS-20, IRI, and NEO-FFI scores by sex were also analyzed (Table 6). All the mean scores of IRI and N, E, O, and A of NEO-FFI were higher for females than for males, except for the EOT score of TAS-20, which was higher for males.

**Table 6:** LEAS-J and TAS-20, IRI, and NEO-FFI score gender differences

	men (*n *= 121)		women (*n *= 223)		*t *value	
	*M*	(*SD*)	*M*	(*SD*)		
[LEAS-J]						
Self	36.95	(11.39)	42.87	(10.19)	-4.94	***
Other	34.25	(9.74)	38.11	(9.15)	-3.65	***
Total	46.71	(10.23)	51.87	(8.37)	-5.03	***
[TAS-20]						
DIF	10.56	(5.75)	11.33	(6.24)	-1.12	
DDF	13.95	(3.64)	14.55	(4.11)	-1.35	
EOT	19.28	(3.93)	18.32	(4.03)	2.13	*
Total	43.79	(9.02)	44.20	(10.28)	-.36	
[IRI]						
FS	16.28	(5.63)	19.04	(5.61)	-4.34	***
PT	15.14	(4.02)	16.43	(4.22)	-2.75	**
EC	16.21	(4.30)	18.22	(4.52)	-4.01	***
PD	15.08	(4.92)	17.24	(5.22)	-3.73	***
[NEO-FFI]						
N	31.41	(7.56)	34.39	(8.02)	-3.35	***
E	28.46	(6.82)	30.57	(7.37)	-2.60	**
O	27.72	(5.84)	29.63	(5.47)	-3.02	**
A	24.47	(6.09)	26.98	(6.33)	-3.55	***
C	29.62	(6.41)	30.76	(7.37)	-1.49	

* *p *< .05 ** *p *< .01 *** *p *< .001

A one factor analysis of variance (ANOVA) was completed for the comparisons based on socioeconomic status. The effect of the parents' position in the job hierarchy on the subjects' LEAS-J score was investigated. No significant effect on the LEAS-J score was observed for the father's or mother's occupation (Table 7).

**Table 7:** LEAS-J total score differences by parents' socioeconomic status

Socioeconomic status by Father's job	High *n *= 85		Middle *n *= 205		Low *n *= 54		*F *value *df *= 2/343	
		
LEAS-J Total score	*Mean*	*(SD)*	*Mean*	*(SD)*	*Mean*	*(SD)*		
	
	48.91	(9.17)	50.49	(9.13)	50.19	(10.61)	0.86	*ns*
**Socioeconomic status by Mother's job**	**High** ***n *= 61**		**Middle** ***n *= 84**		**Low** ***n *= 199**		***F *value** ***df *= 2/343**	
		
LEAS-J Total score	*Mean*	*(SD)*	*Mean*	*(SD)*	*Mean*	*(SD)*		
	
	50.32	(8.47)	50.52	(9.53)	49.77	(9.62)	0.22	*ns*

### Relationship between LEAS-J and other psychological variables

Correlation coefficients were calculated for the mean score of LEAS-J (Self, Other, and Total), TAS-20 (total and each factor), IRI (four scales), and NEO-FFI scores (five factors). Table 8 shows the relationship between LEAS-J and TAS-20, IRI, and NEO-FFI scores. The correlation with TAS-20 showed that only EOT was significantly, negatively correlated with the LEAS-J Self score (r = -.12, p < 0.05).

**Table 8:** Correlation between LEAS-J and TAS-20, IRI, and NEO-FFI

	Self		Other		Total	
[TAS-20]						
DIF	.01		.05		.03	
DDF	-.02		-.01		-.03	
EOT	-.12	*	-.07		-.09	
Total	-.05		.00		-.03	
[IRI]						
FS	.15	**	.12	*	.15	**
PT	.13	*	.13	*	.13	*
EC	.14	**	.15	**	.15	**
PD	.05		.08		.07	
[NEO-FFI]						
N	.08		.06		.07	
E	.17	**	.11	*	.16	**
O	.19	***	.15	**	.20	***
A	.16	**	.17	**	.19	***
C	.04		-.02		.03	

* *p *< .05 ** *p *< .01 *** *p *< .001

With regard to IRI, the LEAS-J Self, Other, and Total scores were significantly, positively correlated with FS (r = .15, p < 0.01; r = .12, p < 0.05; r = .15, p < 0.01, respectively), PT (r = .13, p < 0.05; r = .13, p < 0.05; r = .13, p < 0.05, respectively), and EC (r = .14, p < 0.01; r = .15, p < 0.01; r = .15, p < 0.01, respectively). No significant correlations with PD were observed.

For the NEO-FFI scales, LEAS-J Self, Other, and Total scores were significantly correlated with E (r = .17, p < 0.01; r = .11, p < 0.05, r = .16, p < 0.01), O (r = .19, p < 0.001, r = .15, p < 0.01; r = .20, p < 0.001), and A (r = .16, p < 0.01; r = .17, p < 0.01; r = .19, p < 0.001). No significant correlations were found with N and C.

### Comparisons of the mean scores of LEAS-J and LEAS

We compared the LEAS-J scores with the LEAS scores obtained from a group of Americans students [83 subjects, mean age, 21.12 (2.11) y.o.; male, n = 42; female, n = 41] of the same age. The LEAS-J scores were lower than LEAS scores on all subscales (Self; t[426] = 13.40, p < 0.001, Other; t[426] = 13.31, p < 0.001, Total; t[426] = 11.88, p < .001, Table 9).

**Table 9:** Comparison between LEAS-J scores (Japan) and LEAS scores (U.S.) among students in the same age cohort

	Japan(*n *= 344)		US(*n *= 83)		*t *value	
	*Mean*	*(SD)*	*Mean*	*(SD)*		
Self	40.79	(10.98)	56.33	(9.14)	13.40	***
Other	36.76	(9.53)	51.96	(8.81)	13.31	***
Total	50.05	(9.38)	63.55	(9.12)	11.88	***

*** *p *< .001

### The emotion-related word count of LEAS-J

We extracted 83 samples [males; n = 42, female; n = 41] at random from the present LEAS-J study group to investigate whether or not the emotion-related word count correlates with the LEAS-J scores in the individual protocols. Table 10 shows the correlation coefficients of the emotion-related word count with the LEAS-J scores for each subscale. Significant correlations were found for all subscales (Self; r = .49, p < 0.001, Other; r = .43, p < 0.001, Total; r = .54, p < .001).

**Table 10:** Correlations between LEAS-J scores and the emotion-related word count (n = 83)

	Emotion-related word count					
	Self		Other		Total	
LEAS-J scores						
Self	.49	***				
Other			.43	***		
Total					.54	***

*** *p *< .001

Gender differences in the emotion-related word count of the LEAS-J scores were also investigated (Table 11). Females more often used emotion-related words in their responses to the LEAS-J protocols than did males (Self; t[81] = 2.66, p < 0.01, Other; t[81] = 1.99, p < 0.10, Total; t[81] = 2.42, p < .05).

**Table 11:** Gender differences of the emotion-related word count of LEAS-J

	men(*n *= 42)		women(*n *= 41)		*t *value	
	*Mean*	*(SD)*	*Mean*	*(SD)*		
Self	13.00	(8.25)	17.72	(7.88)	2.66	**
Other	12.64	(7.06)	15.51	(6.07)	1.99	†
Total	25.25	(14.90)	32.76	(13.31)	2.42	*

† *p *< .10 * *p *< .05 ** *p *< .01

## Discussion

Our preliminary analysis showed very high inter-rater reliability for the scoring of our newly developed Japanese version of the LEAS (LEAS-J). Although the relationship with TAS-20 showed rather poor correlation, except for EOT, the results for NEO-FFI and IRI indicate that the LEAS-J has good concurrent validity. The current findings confirm that the Japanese written descriptions of each scenario are essentially equivalent to the original, which indicates that the LEAS-J would be useful for assessing the level of emotional awareness of Japanese subjects.

The LEAS-J was shown to have good internal consistency. The finding for α coefficients was very similar to the findings of a previous study by Lane, et al. [6], suggesting that the LEAS-J provides sufficient reliability. We strictly classified our subjects' descriptions, using the glossary of words in the LEAS scoring manual [24].

In the age cohort comparison of LEAS-J and LEAS scores, however, the mean scores of almost all of the LEAS-J scenes were significantly lower than those of the LEAS. This could be due to a number of factors. The original LEAS was given to students in a classroom setting, while the LEAS-J subjects were requested to complete the scenarios at home. The difference in the collection method might have had some influence on the difference. Another factor may be the Japanese style of expressing feelings, which has been reported to be much different from that of people in Europe and the U.S. [26]. Japanese are said to generally use fewer emotion related words and, in fact, the emotion-related word count of LEAS-J was significantly correlated with the LEAS-J score for every scenario for Self, Other, and Total in the current study. Another possible explanation is that expressing feelings depends on social context: Japanese, in general, tend to attenuate and control emotional expressions more than Americans [27]. Such a tendency modulates the social desirability in the style of expressing feelings, especially when writing; for example, a mature Japanese person should express feeling in a rather reserved way. Perhaps consciously accessible thoughts and rules about what is appropriate directly influence which emotions do and do not reach conscious awareness [28]. It will be necessary in the future to determine if the Japanese style of expressing feelings contributed to the lower LEAS-J scores.

In addition to scoring by the glossary of the original LEAS as stated above, even after the same subjects' words and descriptions were re-scored assessing the individual level of emotional awareness as a whole at the conceptual level, the mean scores of all scenes on the LEAS-J remained significantly lower than the level of the mean LEAS scores. Nisbett & Masuda [29] compared the context sensitivity of Americans and Japanese, and suggested that Asians appear to attend more to the field and Westerners more to salient objects. Further study will be necessary to clarify the differences in the inventories; especially the different styles of emotional life between Americans and Japanese.

Construct and concurrent validity were obtained by examining the relationship between LEAS-J scores and TAS-20, IRI, and NEO-FFI scores and are as follows.

The LEAS-J Self score showed a significant, negative correlation only with the EOT scores of TAS-20. This finding is similar to the finding that the LEAS subscales were negatively correlated with only the EOT of patients with somatoform disorders [30,31] and the finding that in other studies the LEAS tends to correlate most highly with the EOT factor of the TAS-20. In contrast to the DIF and DDF of TAS-20, EOT is more accurate in ratings because the items of EOT ask subjects to rate themselves on a skill or habit that they could easily be aware of [30]. EOT is also less influenced by depression or anxiety [32]. Furthermore, EOT has been more closely associated than the other two factors in various objective measurements regarding affect regulation, such as physiological indices like baseline heart rate [33], and in the Affect Consciousness Interview [34]. Based on these observations, the more specific relation between LEAS-J and EOT should increase the validity of LEAS-J as a psychological measure that can objectively probe an individual's levels of emotional awareness.

The present study is the first report of the relationships between LEAS-J and IRI and NEO-FFI. The significant correlations of all aspects of LEAS-J with fantasy (FS), perspective taking (PT), and empathic concern (EC) in IRI suggest that the ability to take into account the feelings of oneself and others with sensitivity underpins the imaginative function that places others in the position of oneself (FS), an ability associated with understanding the inner emotional state (PT) of others and of consideration for others (EC).

The LEAS-J scales were significantly associated with the NEO-FFI scores for Extraversion (E), Openness-to-Experience (O), and Agreeableness (A). Extraversion includes sociability, liveliness, and the general experience of positive affect. Openness-to-Experience includes aesthetic sensitivity, intellectual curiosity, need for variety, non-dogmatic attitudes, high curiosity and interest in the internal and external world. These positive associations with E and O are consistent with their negative associations with TAS-20 [14,35]. In addition, the present findings for Agreeableness (A) are supported by our previous study showing its negative associations with TAS-20 among Japanese subjects [14]. Agreeableness includes a high degree of a sense of cooperation with others, such as trust, altruism, and sympathy. It appears that in Japan higher emotional awareness contributes to greater agreeableness, which seems consistent with the hypothesis that sensitivity to the others is highly valued in many Asian cultures. On the other hand, no significant correlations were observed between an excessive interest in the negative feelings of oneself (neuroticism) with the Self, Other, or Total LEAS-J scores. This observation confirms evidence that the LEAS is not influenced by depression or a state of anxiety in healthy subjects [13,30,34] and stands in contrast to findings that the TAS-20 is significantly correlated with neuroticism [14,22,36].

The scores of women were higher than those of men for all of the LEAS-J subscales. This is consistent with the report by Barrett, et al. [37], and it is evident that the gender-related difference of emotional awareness is present cross-culturally. The LEAS-J scores were well correlated with the IRI scores in the present study, showing that females tend to be more aware of their own and other's emotions than males. Actually, the emotion-related word count of females on LEAS-J was significantly higher than that of males in the current study, similar to the findings from Barrett et al. [37]. In addition, current findings of gender differences are confirmed by IRI scores [38] and the EOT scores of TAS-20 [14]. Given that this gender difference in emotional awareness has been demonstrated in children as young as 10 years old [39] and early adolescents 12~15 years old [40], these findings raise the distinct possibility that a sex difference in emotional awareness is a biological universal.

In this study, the LEAS-J scale scores were not significantly different by socioeconomic status. However, Lane, et al. [13,34] found significant differences in LEAS scores between groups of differing socioeconomic status. Our findings indicated very similar socioeconomic status for our participants, as might be expected in a homogeneous sample of Japanese undergraduate students, and, indeed, the vast majority of the subjects belonged to the middle class based on the occupational prestige scores [11]. Therefore, we were not able to precisely assess the influence of socioeconomic status on the level of emotional awareness because of the constricted range in our sample. Further, because all subjects were university students who had passed entrance examinations of similar difficulty, it is possible that their comparability in linguistic ability had an equalizing influence on LEAS-J scores.

Further work is necessary in the following areas: LEAS-J should be given to subjects with a wider range of ages to examine the possible influence of age as an indicator of emotional development on LEAS-J scores; to determine if LEAS-J adequately detects impairments of emotional awareness in clinical samples, such as patients with alexithymia; to develop an additional scoring system that is optimally suited to the Japanese language and culture to supplement the current glossary that is derived from LEAS protocols completed by subjects in the United States and is based on a direct translation from English; and finally, the reliability and validity of LEAS-J will need to be examined carefully by a concurrent structured interview, such as the SIBIQ [41].

## Conclusions

A Japanese version of LEAS, LEAS-J, was developed and its reliability and validity were examined. Although a Japanese specific glossary or a modified scoring method will be helpful in the future to capture culture-specific aspects of emotional life in Japan, the current results showed high inter-rater reliability and internal consistency. Construct and concurrent validity were supported by the relationships with TAS-20, IRI, and NEO-FFI. Women had higher scores than men on LEAS-J, supporting the cross-cultural consistency of this gender difference.

## Competing interests

The authors declare that they have no competing interests.

## Authors' contributions

TI collected and analyzed the data, performed the statistical analysis and drafted the manuscript. GK participated in the study design, analysis and interpretation of data and revision of the manuscript. RD made substantive contributions to the study design and interpretation of the results. YM, HN participated in the design of the study and collected the data. HA analyzed the data and YT provided advice on the data analysis. CS participated in the translation of the Japanese version and checked the revised scenarios. MM participated in the acquisition of data and made substantive contributions to the study design. All authors have read and approved the final manuscript
